# Screening HIV-positive men who have sex with men for hepatitis C re-infection risk: is a single question on condom-use enough? A sensitivity analysis

**DOI:** 10.1186/s12879-019-4456-7

**Published:** 2019-09-18

**Authors:** Patrizia Künzler-Heule, Sandra Engberg, Manuel Battegay, Axel J. Schmidt, Katharina Fierz, Huyen Nguyen, Agnes Kocher, Christiana Nöstlinger, Benjamin Hampel, Marcel Stöckle, Charles Béguelin, Julie Delaloye, Patrick Schmid, Markus Flepp, Mathieu Rougement, Dominique Laurent Braun, Jan Fehr, Dunja Nicca, the Swiss HIV Cohort Study (SHCS), A. Anagnostopoulos, M. Battegay, E. Bernasconi, J. Böni, D. L. Braun, H. C. Bucher, A. Calmy, M. Cavassini, A. Ciuffi, G. Dollenmaier, M. Egger, L. Elzi, J. Fehr, J. Fellay, H. Furrer, C. A. Fux, H. F. Günthard, D. Haerry, B. Hasse, H. H. Hirsch, M. Hoffmann, I. Hösli, M. Huber, C. R. Kahlert, L. Kaiser, O. Keiser, T. Klimkait, R. D. Kouyos, H. Kovari, B. Ledergerber, G. Martinetti, B. Martinez de Tejada, C. Marzolini, K. J. Metzner, N. Müller, D. Nicca, P. Paioni, G. Pantaleo, M. Perreau, A. Rauch, C. Rudin, A. U. Scherrer, P. Schmit, R. Speck, M. Stöckle, P. Tarr, A. Trkola, P. Vernazza, G. Wandeler, R. Weber, S. Yerly

**Affiliations:** 1 Nursing Science, Department Public Health, Faculty of Medicine, University of Basel, Bernoullistrasse 28, CH-4056 Basel, Switzerland; 2 Department of Gastroenterology/Hepatology and Department of Nursing Development, Cantonal Hospital St. Gallen, St. Gallen, Switzerland; 3 University of Pittsburgh, School of Nursing, Pittsburgh, PA USA; 4 Division of Infectious Diseases and Hospital Epidemiology, University Hospital Basel, Basel, Switzerland; 5 Medical Faculty, University of Basel, Basel, Switzerland; 6 Division of Infectious Diseases, Cantonal Hospital St. Gallen, St. Gallen, Switzerland; 7 Sigma Research, London School of Hygiene and Tropical Medicine, London, UK; 8 Zurich University of Applied Sciences (ZUAS), Winterthur, Switzerland; 9 Division of Infectious Diseases and Hospital Epidemiology, University Hospital Zurich, Zurich, Switzerland; 10 Institute of Medical Virology, University of Zurich, Zurich, Switzerland; 11 Department of Public Health, Institute of Tropical Medicine, Antwerp, Belgium; 12 Department of Applied Psychology, University of Wien, Vienna, Austria; 13 Department of Infectious Diseases, Bern University Hospital and University of Bern, Bern, Switzerland; 14 Department of Intensive Care Medicine, University of Lausanne and University Hospital, Lausanne, Switzerland; 15 Center for Infectious Diseases, Klinik im Park, Zurich, Switzerland; 16 Primary Care Medicine Unit, University Hospital of Geneva, Geneva, Switzerland; 17 Department of Public Health, Epidemiology, Biostatistics and Prevention Institute, University of Zurich, Zurich, Switzerland; 18 Ressort MTT, University Hospital Basel, Basel, Switzerland

**Keywords:** HIV, Hepatitis C virus, Homosexuality, Male, Sexual behavior, Condoms

## Abstract

**Background:**

Hepatitis C virus (HCV) is common in men who have sex with men (MSM) with HIV. The Swiss HCVree Trial targeted a micro-elimination by using a treat and counsel strategy. Self-reported condomless anal intercourse with non-steady partners was used as the selection criterion for participation in a counselling intervention designed to prevent HCV re-infection. The purpose of this study was to assess the ability of this criterion to identify men who engaged in other sexual risk behaviours associated with HCV re-infection.

**Methods:**

Men who disclosed their sexual and drug- use behaviours during the prior 6 months, at study baseline, were included in the current study. Using a descriptive comparative study design, we explored self-reported sexual and drug-use risk behaviours, compared the odds of reporting each behaviour in men who reported and denied condomless anal intercourse with non-steady partners during the prior year and calculated the sensitivity/specificity (95% CI) of the screening question in relation to the other at-risk behaviours.

**Results:**

Seventy-two (61%) of the 118 men meeting eligibity criteria reported condomless anal intercourse with non-steady partners during the prior year. Many also engaged in other potential HCV transmission risk behaviours, e.g., 52 (44%) had used drugs. In participants disclosing drug use, 44 (37%) reported sexualised drug use and 17 (14%) injected drugs. Unadjusted odds ratios (95% CI**)** for two well-known risk behaviours were 2.02 (0.80, 5.62) for fisting and 5.66 (1.49, 37.12) for injecting drug use. The odds ratio for sexualised drug use - a potential mediator for increased sexual risk taking - was 5.90 (2.44, 16.05). Condomless anal intercourse with non-steady partners showed varying sensitivity in relation to the other risk behaviours examined (66.7–88.2%).

**Conclusions:**

Although condomless anal intercourse with non-steady partners was fairly sensitive in detecting other HCV relevant risk behaviours, using it as the only screening criterion could lead to missing a proportion of HIV-positive men at risk for HCV re-infection due to other behaviours. This work also points to the importance of providing access to behavioral interventions addressing other sexual and drug use practices as part of HCV treatment.

**Trial registration:**

Clinical Trial Number: NCT02785666, 30.05.2016.

## Background

In men who have sex with men (MSM) living with HIV, co-infection with hepatitis C virus (HCV) has become a concern over the last 20 years [1]. An HCV RNA-screening of MSM with HIV (*n* = 3722) participating in the Swiss HIV Cohort Study (SHCS) between October 2015 and May 2016 showed a prevalence of 4.8% (*n* = 177) [2]. People living with an HIV/HCV co-infection show faster progression of liver fibrosis compared to people with HCV mono-infection and higher risk for liver-related morbidity and mortality [3]. Since the introduction of the new direct acting antivirals (DAAs) cure is possible in 95% of the cases, making micro-elimination of HCV a realistic target [4]. However, the population of MSM with HIV frequently present with HCV (re-) infections and current evidence shows that sexual transmission is one important source of (re-) infection [5]. Addressing sexual risk behaviour should become an essential component of HCV medical treatment [6].

In MSM, several sexual behaviours have been described as potentially risky, for example mucosally traumatic sexual behaviours including condomless anal intercourse (CAI), receptive fisting, rectal bleeding, anal douching, sharing of sex toys and group sex activities; nasally applied drugs; injection drug use and drug use in combination with sex [7–9]. Still, to-date, it remains controversial which risk behaviours are the most important ones regarding HCV transmission in MSM with HIV, and should subsequently constitute the most important targets for preventive efforts [10].

From 2015 to 2017, the Swiss HCVree Trial was conducted as an investigator-initiated substudy of the SHCS using a test, treat and counsel strategy with the goal to eliminate HCV in the MSM population with HIV [11]. An E-health assisted behavioural counselling intervention with nurses as counselors was developed and implemented with the aim to reduce sexual risk taking. MSM co-infected with HIV/HCV were asked to participate in the counseling intervention if they reported condomless anal intercourse with non-steady partners (nsCAI) the year prior to starting treatment [11]. Condomless anal intercourse was the only risk behaviour for which SHCS data was available [12] at the time of intervention development. However, its usefulness in selecting participants for the additional sexual risk reduction intervention remains questionable given that other sexual and drug-using behaviours are also important risk factors for HCV transmission. The current analysis was conducted to investigate the usefulness of nsCAI as the selection criterion for the behavioural intervention. This can provide important information for further studies. Specifically, the aims of this study were to (1) describe sexual and drug-using behaviours participants reported during Swiss HCVree study baseline assessment and to compare those behaviours in MSM who did and did not report nsCAI during the prior year and to (2) examine the condom-use question’s sensitivity and specificity in identifying men who engaged in other HCV relevant risk behaviours and who may, therefore, also benefit from risk reduction interventions.

## Methods

A descriptive comparative study design was used to address the objectives and included a comprehensive assessment of social, medical and behavioural factors. Data were compared for differences between the two groups: those who reported nsCAI and those who denied nsCAI during the prior year.

### Setting and participants

The Swiss HCVree Trial was implemented within the framework of the SHCS, an ongoing multicentre prospective observational study that started in 1988. Its participants have been shown to be highly representative of all known people living with HIV (PLWH) in Switzerland, [13] and modelling studies estimate that 84% of all MSM with HIV in Switzerland are followed in the SHCS [14]. During the Swiss HCVree Trial (2015–2017), all adult men with self-identified homosexual or bisexual preferences enrolled in the SHCS (*n* = 3722) were assessed for HCV ribonucleic acid (RNA) [2]. One hundered twenty-two (122) were diagnosed with HCV and treated with DAAs in one of eight specialized HIV clinics in Switzerland [11] and all but one individual were cured. Among the men treated with DAAs, a positive response to the nsCAI question in the SHCS during the prior year was used to select men who were invited to participate in the sexual risk reduction intervention performed by nurses.

### Data collection

The data used in this analysis were retrieved from the SHCS database and the Swiss HCVree Trial baseline assessment. Data included sociodemographic characteristics (age, ethnicity/race, highest completed educational degree) and medical information about HIV from the SHCS database and HCV specific information from the Swiss HCVree Trial. At Swiss HCVree Trial baseline, participants were asked to complete a self-reported questionnaire about sexual and drug-use behaviours during the previous 6 months. Table 1 summarizes the data collected.

**Table 1:** Data collected

Database	Domain Variables assessed	Question	Answer
SHCS, reported in interview situation	Screening question		
	Selection criteria for sexual risk reduction intervention	“Over the last 12 months, did you have unprotected anal intercourse with occasional partners?”	Yes/no
Swiss HCVree Trial, Self-completed questionnaires	Sociodemographic		
	Partnership	“Did you have a stable partnership in the last 6 months?”	Yes/no
	Risk Behaviours		
	Sextoys	“Over the last 6 months, did you use sextoys with non-steady partners?”	Yes/no
	Fisting	“Over the last 6 months, did you practice fisting?”	Yes/no
	Drug use	“Did you use one or more of the following substances in the last 6 months?”	
		Cocaine	Yes/no
		γ-butyrolactone/γ-hydroxybutyric acid (GHB/GBL)	Yes/no
		crystal methamphetamine (CM)	Yes/no
		ketamine	Yes/no
		mephedrone	Yes/no
		“If your answer is yes, how did you take the substance(s)?”	injection (slammed)/ intranasal/orally/smoked/ mucosally (anal)
	Sexualised drug use	“If your answer is yes, did you take any of the above-mentioned substance(s) in combination with sex?”	Yes/no
	Psychological constructs		
	Attitudes towards condom use	Sexual risks scale-attitudes toward condom use [15] 13 items rated on a 5-point Likert scale	1 (I don’t agree at all) to 5 (I completely agree). Possible scores range from 13 to 65
	Condom self-efficacy	Self-efficacy for negotiating condom use [16], 5 items rated on a 1–10 scale	0 (I cannot) to 10 (I am sure that I can). Possible scores range from 0 to 50

### Data analysis

Analyses were conducted using the open source R statistical analysis software (Version 1.0.136 for Mac OS X). Participants’ characteristics and self-reported at-risk sexual and drug-use behaviours were analysed descriptively. Depending on the level of measurement and distribution of variables, frequencies, percentages, means and standard deviations (SD), or median and interquartile range (IQR) were calculated. Based on the SHCS data, participants were divided into two groups: those who reported no sex with non-steady partners or only protected anal intercourse during all sexual encounters during the last 12 months (i.e. without nsCAI) and those reporting nsCAI. Baseline characteristics, attitudes and self-efficacy regarding condom use were compared in the two nsCAI groups. Chi-square tests were used to compare categorical variables and the student’s t-test (for age, which was normally distributed) or Mann-Whitney U tests (for years since HIV diagnosis and scores on the attitudes toward condom use and self-efficacy questionnaires, which were not normally distributed) were utilised to compare continuous variables. Odds ratios and their 95% confidence intervals (CI) were calculated to examine the association between nsCAI and the other risk behaviours assessed. Multivariable logistic regression was conducted to determine if adjusting for age and duration of HCV affected the relationship between nsCAI and the other risk behaviors. We used a manual stepwise backward elimination. MedCalc online software (https://www.medcalc.org/calc/diagnostic_test.php) was used to calculate the sensitivity and specificity (including 95% CI) of the condom use screening question with non-steady sexual partners in relation to the other at-risk sexual and drug use behaviours.

## Results

**Fig. 1:**
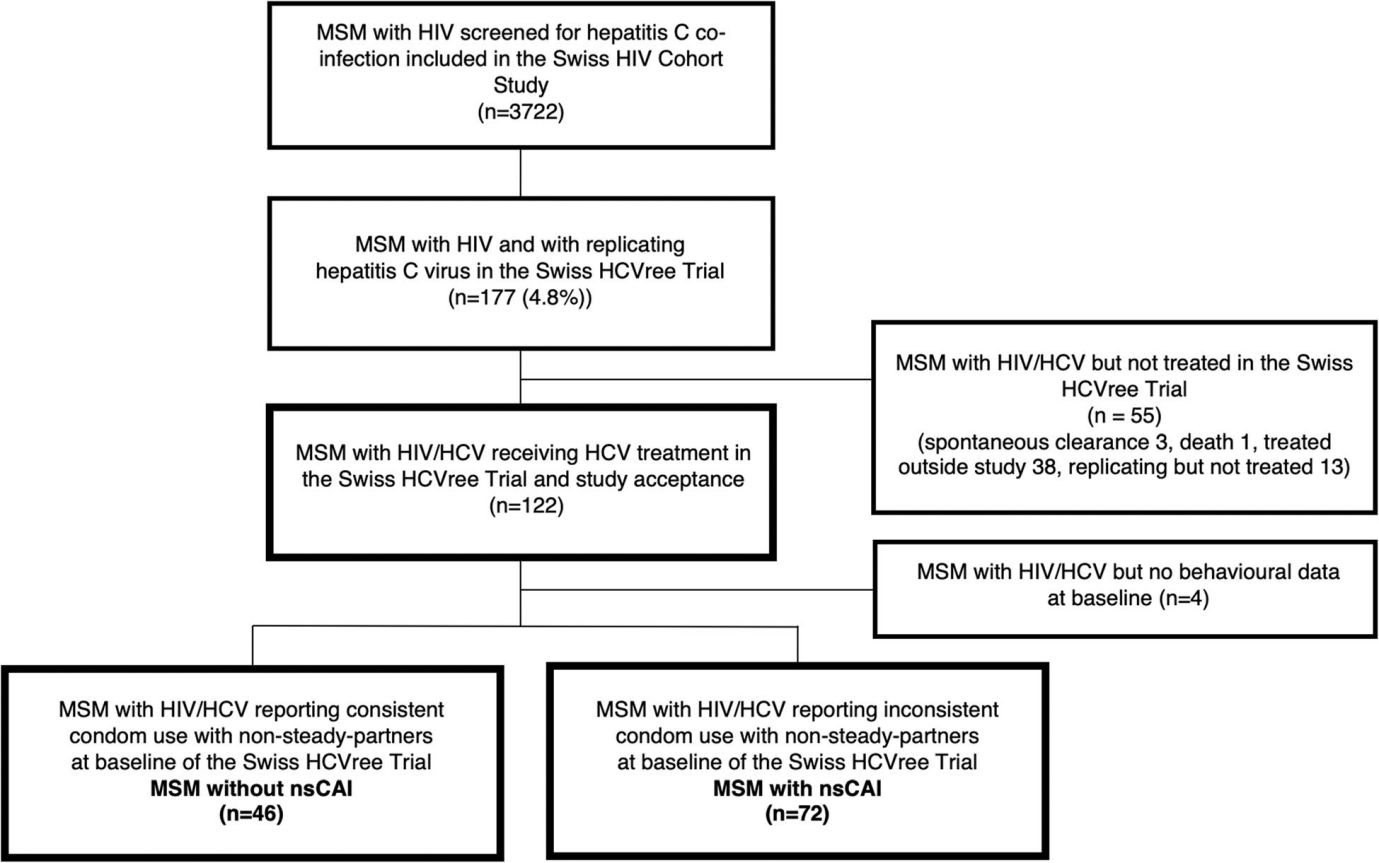
Flowchart Swiss HCVree Trial and group building according to men’s response to the nsCAI screening question

During the Swiss HCVree Trial baseline assessment, 118 of 122 participants disclosed their sexual and drug-use behaviours and were included in the current study, see Fig. 1.

Based on SHCS data, 72 (61%) MSM reported nsCAI and 46 (39%) reported no nsCAI during the 12 months prior to enrollment in the Swiss HCVree Trial. There were no significant differences in the two groups’ socio-demographic characteristics. There were significant group differences in the years since HCV diagnosis; MSM with nsCAI had a shorter median duration of 1.9 years (0.9–5.1) compared to MSM without nsCAI with a median duration of 4.8 years (2.1–10.3). Participants without nsCAI scored significantly more positive attitudes toward condom use and had higher self-efficacy related to condom use than men with nsCAI (median score = 44.00 vs. 39.00, *p* = .023 and median score = 40.72 vs. 29.23, *p* < .001 respectively).

Many men reported engaging in a variety of sexual or drug-use behaviours identified as risk factors for HCV-infection: 25 (24%) shared sextoys, 28 (25%) practised fisting and 52 (44%) used drugs during the prior 6 months. In participants who answered the drug-use questions, 44 (37%) reported sexualised drug use and 17 (15%) injected drugs. Participants reported using the following drugs: 30 (26%) used γ-butyrolactone/γ-hydroxybutyric acid (GHB/GBL), 26 (22%) cocaine, 22 (19%) crystal methamphetamine, 11 (9%) ketamine and 10 (9%) mephedrone (Table 2). Those with nsCAI during the 12 months prior to treatment were more likely to have engaged in other risky sexual behaviours than those without nsCAI although the odds in the two groups were only statistically significant for drug use, drug use during sex and injecting drugs. Adjusting for age and/or HCV duration did not change the relationship between nsCAI and the other risk behaviours examined in terms of the direction or significance of the odds ratios.

**Table 2:** Sociodemographic and HCV-related risk behaviours in the last 6 months at study baseline

Sociodemographic and HCV-related risk behaviours at study baseline	Total (*n* = 118)	Participants without nsCAI (*n* = 46)	Participants with nsCAI (*n* = 72)	Univariable Odds Ratio (95% CI)	Multivariable Adjusted OR (95% CI) for age and HCV duration	Adjusted OR (95% CI) for HCV duration
Age, mean (sd)	46.6 (+/− 9.2)	49.0 (+/− 9.1)	45.1 (+/− 9.1)	0.64 (0.41, 0.96)^a^		
HCV duration, median (IQR)	2.9 (1.1–7.1)	4.8 (2.1–10.3)	1.9 (0.9–5.1)	0.87 (0.80, 0.94)		
Sharing sextoys, n (%) (*n* = 104/38/66)^b^	25 (24)	7 (18)	18 (28)	1.53 (0.58, 4.40)	1.05 (0.36, 3.21)	1.08 (0.37, 3.29)
Fisting, n (%) (*n* = 114/43/71)^b^	28 (25)	7 (16)	21 (30)	2.02 (0.80, 5.62)	2.12 (0.78, 6.31)	1.92 (0.72, 5.60)
Drug use, n (%) (*n* = 117/45/72)^b^	52 (44)	8 (18)	44 (61)	7.27 (3.08, 18.91)	5.58 (2.26, 15.02)	5.79 (2.37, 15.42)
GHB/GBL, n (%)	30 (26)	3 (7)	27 (38)	8.60 (2.78, 37.87)	6.64 (2.04, 30.18)	6.91 (2.15, 31.07)
Cocaine, n (%)	26 (22)	6 (13)	20 (28)	2.56 (0.99, 7.55)	2.36 (0.85, 7.39)	2.49 (0.91, 7.6)
Crystal methamphetamine, n %)	22 (19)	1 (2)	21 (29)	18.48 (3.63, 338.0)	15.47 (2.89, 288.31)	15.91 (3.01, 294.78)
Ketamine, n (%)	11 (9)	2 (4)	9 (14)	3.12 (0.76, 21.14)	3.55 (7.82, 25.71)	3.55 (7.82, 25.71)
Mephedrone, n (%)	10 (9)	–	10 (15)			
Use of any of the drugs listed above during sex, n (%) (*n* = 116/45/71)^b^	44 (38)	7 (16)	37 (52)	5.90 (2.44, 16.05)	4.42 (1.73, 12.52)	4.63 (1.84, 12.92)
Reporting injection of drugs, n (%) (*n* = 117/45/72)^b^	17 (15)	2 (4)	15 (21)	5.66 (1.49, 37.12)	4.45 (1.10, 30.15)	4.53 (1.13, 30.51)

^a^Unit 10 years

^b^specified how many HIV-positive MSM answered the question (n = total group/without nsCAI/with nsCAI)

Odds ratios for two sexual behaviours with established transmission risk were 2.02 (0.80, 5.62) for fisting and 5.66 (1.49, 37.12) for injecting drug use. Sexualised drug use, a potential mediator for increasing other risk behaviours, showed an odds ratio of 5.90 (2.44, 16.05), see Table 2.

Table 3 summarizes the results of analyses examining the sensitivity and specificity of reporting consistent condom-use with non-steady partners at study baseline in identifying men who did not engage in the other at-risk behaviours examined. The nsCAI question had the highest sensitivity in relation to the question about injecting drugs (88.2%) and lowest for sharing sex toys (66.67%). Specificity was low in all analysed risk behaviours (41.18–57.58%).

**Table 3:** Sensitivity analysis of screening question “nsCAI” to identify other probable risk behaviours for HCV re-infection

Risk Behaviours	Sensitivity^a^ (%) (95% CI)	Specificity^b^ (%) (95% CI)
Any drug use	84.62 (71.92–93.12)	57.58 (44.79–69.66)
Sexualised drug use	84.09 (69.93–93.36)	52.70 (40.75–64.43)
Injecting drug use	88.24 (63.56–98.54)	43.56 (33.72–53.80)
Fisting	75.00 (55.13–89.31)	43.18 (32.66–54.18)
Sharing of sex toys	66.67 (48.17–82.04)	41.18 (30.61–52.38)

^a^The probability that HIV-positive MSM report a selected risk behaviour will also report nsCAI

^b^The probability that HIV-positive MSM will deny nsCAI if they are not engaging in other selected other risk behaviours

## Discussion

The MSM co-infected with HIV/HCV in this study practiced various sexual and drug use behaviours associated with HCV transmission risk in addition to condomless sex. While nsCAI was associated with higher odds of engaging in other behaviours, based on our findings, relying only on this question to identify men at risk for HCV re-infection is likely miss a proportion of MSM with HIV at risk for HCV due to other behaviours. Between 16 to 18% of the men who denied nsCAI reported engaging in other behaviors that have been associated with an increased risk of HCV re-infection. Eighteen percent (18%) of those who denied nsCAI reported using drugs. This is an important finding as drug use is seen as a potential mediator for increased sexual risk-taking [17, 18].

Condom use was the only risk behaviour available for all men in the SHCS and was for this reason used as the criterion for selecting men to participate in the sexual risk reduction behavioural intervention portion of the Swiss HCVree Trial [19]. Despite our use of this inclusion criterion, its discriminatory value in identifying men at high risk for HCV re-infection was unclear. However, a recent study from London found that CAI was a significant risk factor for acute HCV infection in MSM and in one third of participants it was the only risk factor [9]. In contrast to our study, MSM received care in a sexual health clinic and benefitted from a multi-disciplinary prevention approach including harm reduction services whereas in our study, HCV treatment was given in specialised medical HIV clinics. In line with other investigations in MSM with HIV, study participants reported various behaviours other than nsCAI that potentially increased their risk of HCV sexual transmission [9]. It has been well documented that condoms are less attractive in the MSM community – largely due to the common understanding and awareness that HIV treatment is preventive in terms of HIV transmission [20]. Decreasing trends of condom use was confirmed in a systematic review of studies across high-income countries [21]. Champenois et al. [22] reported that for MSM with HIV the main reasons for not using condoms were serosorting and being on antiretroviral therapy (ART) with undetectable viral loads. While these traditional HIV-related risk reduction strategies (serosorting and effective HIV treatment) have been shown to prevent the transmission of HIV, they have little or no effect in preventing HCV or other sexually transmitted diseases.

In our study, MSM with HIV and nsCAI were more likely to engage in other risk behaviours compared to those without nsCAI but the relationship was only statistically significant for drug-use and sexualized drug-use. However, due to the small sample size, our study was probably only adequately powered to detect large differences in the groups. They were two-times more likely to practice fisting and six times more likely to report sexualised drug use. The sensitivity of the nsCAI question was 85% in relation to drug use. Nevertheless, our findings indicate that using nsCAI as the only risk behaviour criterion to select men for the behavioural intervention was likely to have resulted in failure to include between 12 and 34% of those engaging in other risk behaviours. Each single behaviour included in the current analysis carries a specific HCV transmission risk; however, which behaviour or combinations of behaviours carry the highest risks is currently less clear and cannot be answered with this study design.

Our results are in line with other studies showing associations between higher rates of drug use/sexualised drug use and risk behaviors [18, 23, 24]. A substantial proportion of our participants reported drug use (44%). Among the men who answered these questions (116 for sexualized drug use and 117 for injecting drugs), 38% reported sexualised drug use and 15% reported injecting substances. In comparison, in two earlier studies on MSM with HIV– one from Madrid (*n* = 742) [23] and one from England/Wales (*n* = 392) [24]– 29.1–29.5% of participants indicated sexualised drug use and 10.1–16% injecting drug use. Our group’s higher rate of sexualised drug use might reflect differences in the study population, especially the fact that our sample’s MSM with HIV were all co-infected with HCV. Several studies have found elevated rates of sexualised drug use in MSM co-infected with HIV/HCV, affirming associations between sexual HCV transmission and higher risk taking behaviours when using substances [25, 26]. Another possible explanation for our group’s high rates of sexualised drug use may be related to the study setting: most of our participants were recruited at the centres in Zurich, a town known for a comparably high prevalence of sexualised drug use. In the European MSM Internet Survey (EMIS-2010), which compared 44 European cities in relation to illicit drug use in MSM, place of residence was the strongest predictor. Zurich reported a 7% prevalence of using one of the four drugs typically used during sex, ranking sixth of the 44 cities studied, just after UK and Spanish cities [8]. In another European survey conducted among MSM in 13 cities, overall prevalence of sex associated with drug use was 11.8% (when measured at the last sexual encounter), and was more frequently reported by MSM with HIV [27].

The four substances typically used during sex were all reported in our study, with GBL/GHB being the most common (25%), followed by crystal methamphetamine (19%). In EMIS (European MSM Internet Survey), percentages of GBL/GHB use were quite similar, but crystal methamphetamine use was lower [8] than in our study, suggesting a surge in its popularity in MSM with HIV. The frequency of cocaine use was also high (22%) – comparable to rates reported in the UK ASTRA trial in MSM with HIV or for Zurich in EMIS [8, 18]. To date, few studies investigating sexualized drug use have included cocaine. However, our results indicate that cocaine may be more common (19%) in sexual contexts than expected.

This study has several limitations. The study’s cross-sectional design precluded any causal inferences about the associations between nsCAI and other behaviours risky for HCV re-infection. During analysis, we identified some limitations in the formulation of questions, e.g., we did not ask about the distinction between insertive or receptive fisting. While self-report questionnaire data may be biased, especially for such sensitive domains as sexual and drug use behaviour, it is often perceived as superior compared to being asked by someone else because of reduced social desirability bias [28]. Given the limited number of MSM co-infected with HIV/HCV in Switzerland, the study sample (118 participants) was small. The small sample size may have limited our ability to detect statistically significant differences in behaviors in the nsCAI and non-nsCAI groups that were clinically meaningful. One strength of the study is that Swiss HCVree Trial (the source of data for this study) screened and treated all participants co-infected with HCV in the SHCS, so the sample is likely to be representative of MSM with HIV living in Switzerland [13].

## Conclusions

Our findings support existing research that MSM co-infected with HIV/HCV engage in various sexual and drug-use behaviours, potentially increasing their risk of HCV re-infection. Men who reported using condoms inconsistently with non-steady partners were more likely to report engaging in the other sexual and drug-use behaviors measured although the differences were only statistically significant for the drug-use behaviors. nsCAI was fairly sensitive in identifying men who also engaged in other risk behaviours, but relying only on it to identify men at risk for HCV infection would miss a proportion of MSM with HIV practicing other potentially modifiable behaviours. Based on our findings we recommend comprehensive screening of potential risk behaviours to identify men whose sexual and drug use behaviors increase their risk for HCV infection. We recommend offering all MSM co-infected with HIV/HCV behavioural interventions designed to reduce sexual and drug use risk behaviours.

## Data Availability

The individual level datasets generated and/or analysed during the current study are not publicly available because open access to all SHCS data is currently not possible. This data is too dense and comprehensive to preserve patient privacy in patients with HIV infection. Free access to the data would currently not be compatible with the SHCS informed consent and with preserving patient privacy. Investigators with a request for selected data should send a proposal to the corresponding author. The provision of data will be considered by the study team and the Scientific Board of the SHCS.

## Abbreviations

CI: Confidence interval
DAAs: Direct acting antivirals
GBL/GHB: γ-butyrolactone/γ-hydroxybutyric acid
HCV: Hepatitis C virus
IQR: Interquartile range
MSM: Men who have sex with men
nsCAI: Condomless anal intercourse with non-steady partners
SD: Standard deviation
SHCS: Swiss HIV Cohort study
STI: Sexual transmitted infections
